# Impact of bronchoscopic thermal vapor ablation on lung volume reduction in patients with emphysema: a meta-analysis

**DOI:** 10.1186/s12890-023-02689-w

**Published:** 2023-10-26

**Authors:** Lijia Zhi, Liping Liao, Zhi Wu, Tiezhu Wang, Yuming Ye, Hao Li, Li Lin, Jia-Chao Qi, Liangji Zhang

**Affiliations:** 1 Department of Intensive Care Unit, Hospital of Chengdu University of Traditional Chinese Medicine, No. 39, Twelve Bridges Rd, Jinniu District, Chengdu, Sichuan Province 610075 People’s Republic of China; 2 Department of Ultrasonic Medicine, Zhangzhou Affiliated Hospital of Fujian Medical University, No. 59, Shengli Rd., Xiangcheng, Zhangzhou, Fujian Province 363000 People’s Republic of China; 3 Department of Respiratory and Critical Care Medicine, Zhangzhou Affiliated Hospital of Fujian Medical University, No. 59, Shengli Rd, Xiangcheng, Zhangzhou, Fujian Province 363000 People’s Republic of China

**Keywords:** Bronchoscopic thermal vapor ablation, Lung volume reduction, Emphysema, Efficacy, Safety

## Abstract

**Background:**

Bronchoscopic lung volume reduction (LVR) could significantly improve pulmonary function and quality of life in patients with emphysema. We aimed to assess the efficacy and safety of bronchoscopic thermal vapor ablation (BTVA) on LVR in patients with emphysema at different stage.

**Methods:**

A systematic search of database including PubMed, Embase and Cochrane library was conducted to determine all the studies about bronchoscopic thermal vapor ablation published through Dec 1, 2022. Related searching terms were “lung volume reduction”, “bronchoscopic thermal vapor ablation”, “bronchial thermal vapor ablation” “BTVA” and “emphysema”, “efficacy” and”safety”. We used standardized mean difference (SMD) to analyze the summary estimates for BTVA therapy.

**Results:**

We retrieved 30 records through database search, and 4 trials were selected for meta-analysis, including 112 patients with emphysema. Meta-analysis of the pooled effect showed that levels of forced expiratory volume in 1 s (FEV1), residual volume (RV), total lung capacity (TLC), 6-min walk distance (6MWD) and St George’s Respiratory Questionnaire (SGRQ) were significantly improved in patients with emphysema following BTVA treatment between 6 months vs. baseline. Additionally, no significant changes in FEV1, RV, TLC and SGRQ occurred from 3 to 6 months of follow-up except for 6MWD. The magnitude of benefit was higher at 3 months compared to 6 months. The most common complications at 6 months were treatment-related chronic obstructive pulmonary disease (COPD) exacerbations (RR: 12.49; 95% CI: 3.06 to 50.99; *p* < 0.001) and pneumonia (RR: 9.49; 95% CI: 2.27 to 39.69; *p* < 0.001).

**Conclusions:**

Our meta-analysis provided clinically relevant information about the impact and safety of BTVA on predominantly upper lobe emphysema. Particularly, short-term significant improvement of lung function and quality of life occurred especially within the initial 3 months. Further large-scale, well-designed long-term interventional investigations are needed to clarify this issue.

**Supplementary Information:**

The online version contains supplementary material available at 10.1186/s12890-023-02689-w.

## Introduction

The morbidity and mortality of chronic obstructive pulmonary disease (COPD) have been still increasing constantly [1]. As a leading characteristic of COPD, predominant emphysema may indicate poor prognosis independent of optimal pharmacological therapy [2]. The therapeutic strategy of COPD focused on relieving symptoms and slowing down the progression of disease. Both surgical and bronchoscopic lung volume reduction (BLVR) have been shown to significantly improve pulmonary function, dyspnea and quality of life in patients with emphysema and hyperinflation [3]. However, the beneficial effects of some LVR procedures maybe temporary [4]. Known as the most comprehensive trial regarding lung volume reduction surgery (LVRS), it reported that benefits can be obtained in patients with emphysema, particularly those with heterogeneous emphysema and upper lobe predominance [5]. Particularly, restricted to high mortality and morbidity rates, LVRS providing substantial long-term clinical benefit was not applied widely in the treatment of patients with severe emphysema [6]. In addition, patients who received LVR should consider the phenotype of emphysema and physiological variables including the stage of disease [7].

It is recommended that BLVR accompanied with reduced mortality and morbidity could be an alternative approach to the LVRS in GOLD guidelines for COPD patients [8]. BLVR was achieved by various techniques such as bronchial valves, endobronchial coils, bronchoscopic thermal vapor ablation (BTVA) and airway bypass [9, 10]. As the most investigated forms of BLVR, data about insertion of intrabronchial valves have documented significant differences in 6-min walk distance (6MWD) and St George’s Respiratory Questionnaire (SGRQ) in the absence of improvement in forced expiratory volume in 1 s (FEV1) [11]. However, it needs to be placed on a lobar basis for valve and coil. Additionally, valve implants do not achieve adequate volume reduction in the presence of collateral ventilation from incomplete fissures [2]. All other methods of BLVR showed efficacy in primary outcomes except for the airway bypass stents. However, in comparison with controls, the sealants showed the most significant findings including FEV1, 6MWD and SGRQ, and it was the least associated with major treatment-related complications [12].

Irrespective of the presence of collateral ventilation, BTVA induced parenchymal thermal damage and inflammation leading to fibrosis and volume reduction in the targeted regions [13, 14]. A clinical trial enrolling 44 patients with upper lobe predominant emphysema demonstrated significant lobar reduction, reduced hyperinflation and improved airflow after BTVA at 6 months [14]. Also, patients were treated unilaterally on lobar level in a more comprehensive single arm trial, showing successful segmental treatment with acceptable safety profile in emphysema patients [15]. Indeed, studies demonstrated that a greater LVR with a lower residual volume (RV) especially in these patients could be observed at 12 months follow-up. Notably, the follow-up period on benefits and safety observed after BTVA varied from 3 to 12 months [14–17]. On the other hand, the most common events were COPD exacerbation and pneumonia among the BTVA-induced adverse effects [18]. However, little was known about whether there were dynamic differences in BTVA-related efficacy and safety at different follow-up stages.

A number of studies were discussed in the need for BTVA in the COPD-related emphysema [14–22]. Therefore, we conducted a meta-analysis to determine the efficacy and safety of BTVA in patients with emphysema.

## Methods

### Materials and methods study selection

This meta-analysis was performed according to the PRISMA guidelines [23]. A systematic search of database including PubMed, Embase and Cochrane library was conducted to determine all the studies about bronchial thermal vapor ablation published through Dec 1, 2022. All searches included free text and corresponding MeSH terms, and the combination of following search terms were used: “lung volume reduction”, “bronchoscopic thermal vapor ablation”, “bronchial thermal vapor ablation” “BTVA” and “emphysema”, “efficacy” and “safety”.

**Fig. 1:**
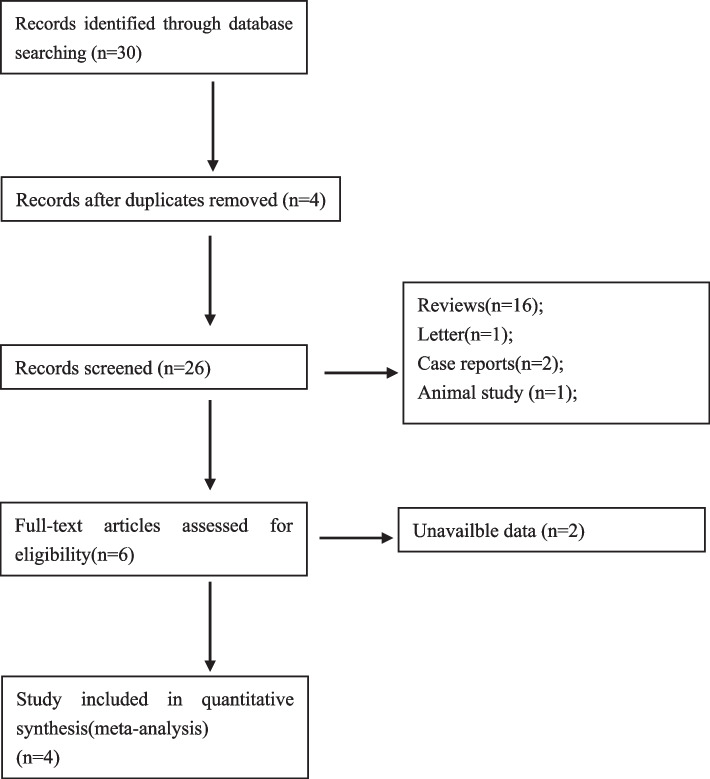
Flow diagram of study selection

To ensure a thorough search of the literature, we conducted manual searches of reference lists from the relevant original and review articles to identify additional eligible studies. And all the abstracts, studies, and citations were reviewed. Prospective nonrandomized and randomized controlled trials (RCTs) providing pre- and post-intervention data (absolute numbers) or mean difference (between pre- and post-intervention) were available. For inclusion in our meta-analysis, only those studies reporting the pre-BTVA and post-BTVA on lung functions including the FEV1, total lung capacity (TLC), RV, 6 MWD and SGRQ were considered. Prospectively conducted multicenter cohort studies with retrospective analyses were also considered eligible for inclusion. No disagreements between investigators on the inclusion or exclusion of a study existed. Figure 1 summarizes the results of the selection process. As a general rule, for multiple publications of the same trials, we intended to include only the most recent one.

### Inclusion and exclusion criteria

The inclusion criteria were following: (1) observational studies or RCTs; (2) sufficient pre- and post-intervention data were reported allowing for a meta-analysis; (3) study populations were limited to adults (age ≥ 18). (3) subjects with emphysema were diagnosed for the first time and never received any form of treatment before except for BTVA; (4) it was predominant upper-lobe emphysema and a heterogeneity index emphysema from high-resolution computed tomography was (HRCT) > 1.2 [14].

Studies were excluded if they involved: (1) non-English literature; (2) abstracts, expert opinions, case reports, letters, animal studies, editorials and reviews without original data; and (3) unpublished data from conference. The research with the largest population was included if multiple studies reported effects using the same patient group. The corresponding author would be contacted when the studies did not provide adequate data. And the studies would be excluded after two no-response attempts.

### Study outcomes

Primary outcomes including assessments of lung function (FEV1), lung volumes (TLC and RV), exercise capacity (6 MWD), and the health-related quality of life (SGRQ) were reported. And the safety profile was related to BTVA-induced adverse effects.

### Data extraction and analysis

Two reviewers independently extracted data as follows: first author, year of publication, nation, sample size, therapy duration, study design, and outcomes and major adverse events. All the disagreements associated with eligibility were resolved by a third reviewer through discussion until a consensus was reached. Pre- and post-BTVA FEV1, TLC, RV, 6-MWD, and SGRQ with standardized mean difference (SMD), and where necessary, the mean difference with SD or 95% confidence intervals (CIs) were extracted to compare. We used Stata statistical software (Version 12.0, Stata Corporation, College Station, TX, USA) to conduct the meta-analysis. SMD was applied for analyzing the summary estimates. Q and I^2^ statistics were identified as statistical heterogeneity among individual studies. If evidence of statistical heterogeneity indicated by *p* < 0.10 or I^2^ > 50% existed, a randomized-effects model would be applied to combine effect size. Otherwise, we conducted a fixed-effects model to estimate the pooled effects. We performed sensitivity analysis to explore the influence of a single study on overall efficacy of BTVA. Potential publication bias was presented applying funnel plot and tested by “Begg test”. A *p* < 0.05 was identified as statistically significant for the overall effect size.

### Study quality

Two reviewers independently assessed the quality of trials, and they solved any disagreement by consensus. According to the criteria prescribed by the Cochrane Handbook for Systematic Reviews of Intervention, we evaluated the quality of included RCTs based on Cochrane risk of bias [24]. We used RevMan 5.3 (Cochrane Library Software, Oxford, UK) to assess selection bias, performance bias, detection bias, attrition bias, reporting bias and other biases. Three potential types of bias including low risk, high risk, and unclear risk were identified for each single trial during the assessment. A low-risk bias showed that when all the seven items meet the criteria as “low risk”, and a high risk of bias showed that when at least one of the seven items was assessed as “high risk”.

## Results

### Characteristics of selected studies

We retrieved 30 records through database search, some studies did not provide adequate data after BTVA [18–22], and there was no related meta-analysis was registered [22]. Finally, four trials [14–17] were selected for meta-analysis (Fig. 1), including 112 patients with emphysema. According to the criteria discussed previously, all the included trials were deemed to show a low risk of bias. The characteristics of these studies are shown in Table 1. The follow-up of BTVA varied from 3 to 12 months.

**Table 1:** The summary of clinical trials in BTVA therapy

Study	Year	Nation	Sample size	Therapy duration	Study design	Outcome	Major adverse events
Zarogoulidis, P [17]	2020	Greece	11	9 months	Observational	Lung Function and quality of life	AECOPD, Infection,
Herth, F. J [15]	2016	Germany	70	6 months	RCT	Lung Function and quality of life	AECOPD Pneumonia or pneumonitis
Snell, G. I [14]	2012	Australia	44	6 months	Open-label, single arm trial	Lung Function and quality of life	Lower respiratory events
Snell, G. I [16]	2009	Australia	11	6 months	Observational	Lung Function and quality of life	pneumonia and exacerbations of airways disease

*RCT* randomized controlled trial, *AECOPD* acute exacerbations of chronic obstructive pulmonary disease

### Effect on primary outcomes

**Fig. 2:**
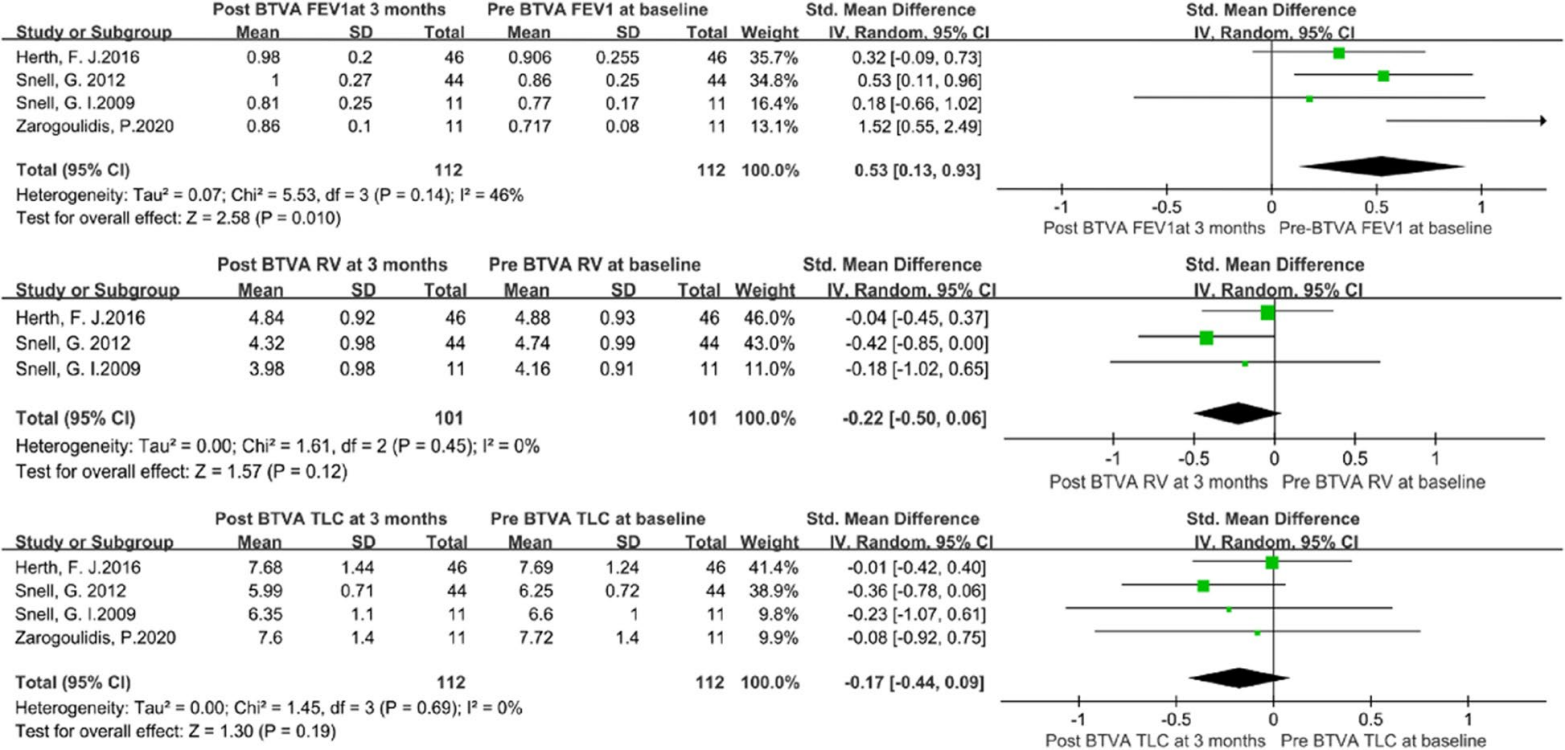
Meta-analysis and forest plot of all studies included about FEV1, RV and TLC between 3 months vs baseline. Calculations based on a randomized-effects model. SMD, standardized mean difference; FEV1, forced expiratory volume in 1 s; RV, residual volume; TLC, total lung capacity

**Fig. 3:**
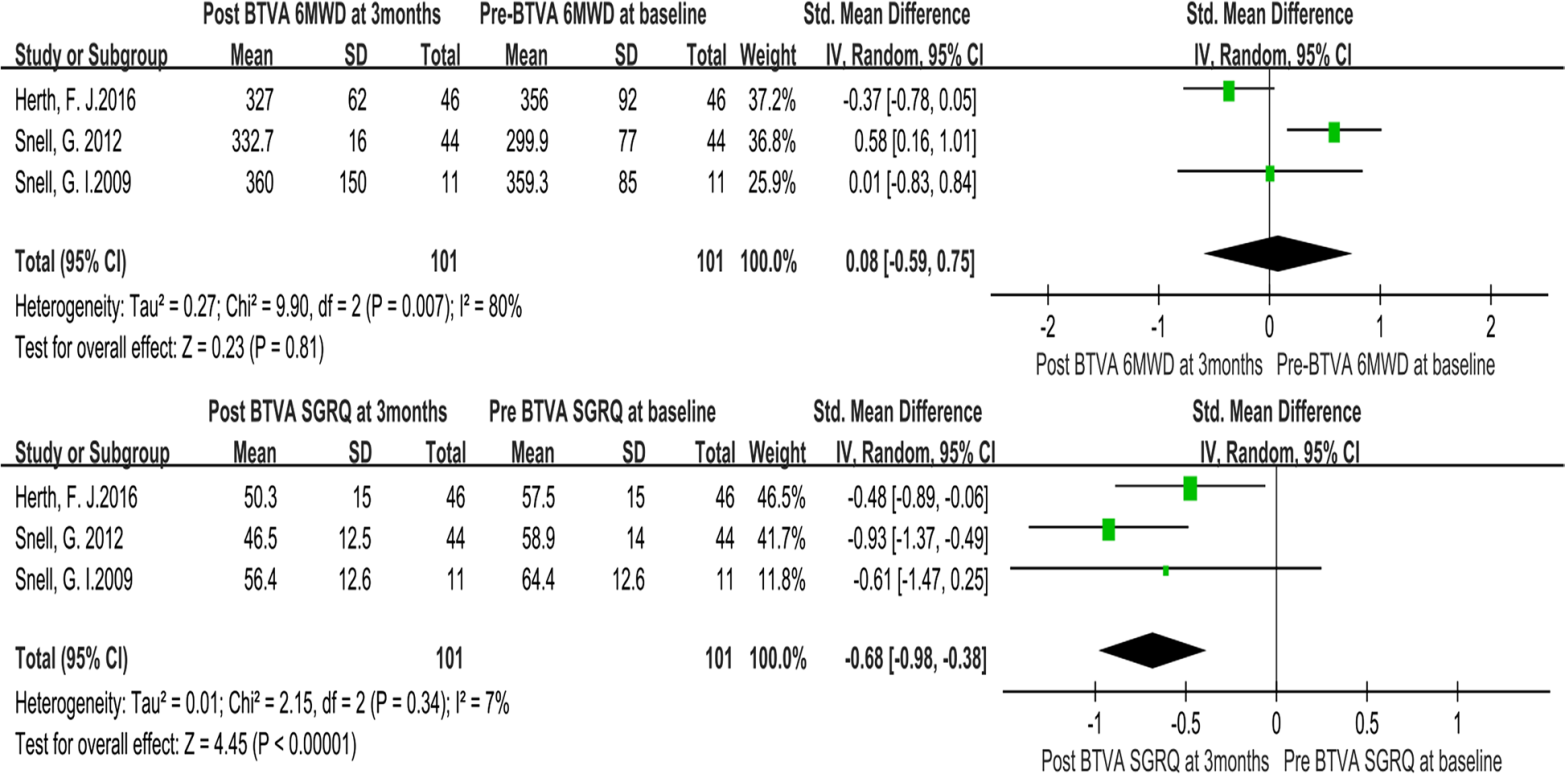
Meta-analysis and forest plot of all studies included about 6MWD and SGRQ between 3 months vs baseline. Calculations based on a randomized-effects model. SMD, standardized mean difference; 6MWD, 6-min walk distance; SGRQ, St George’s Respiratory Questionnaire

**Fig. 4:**
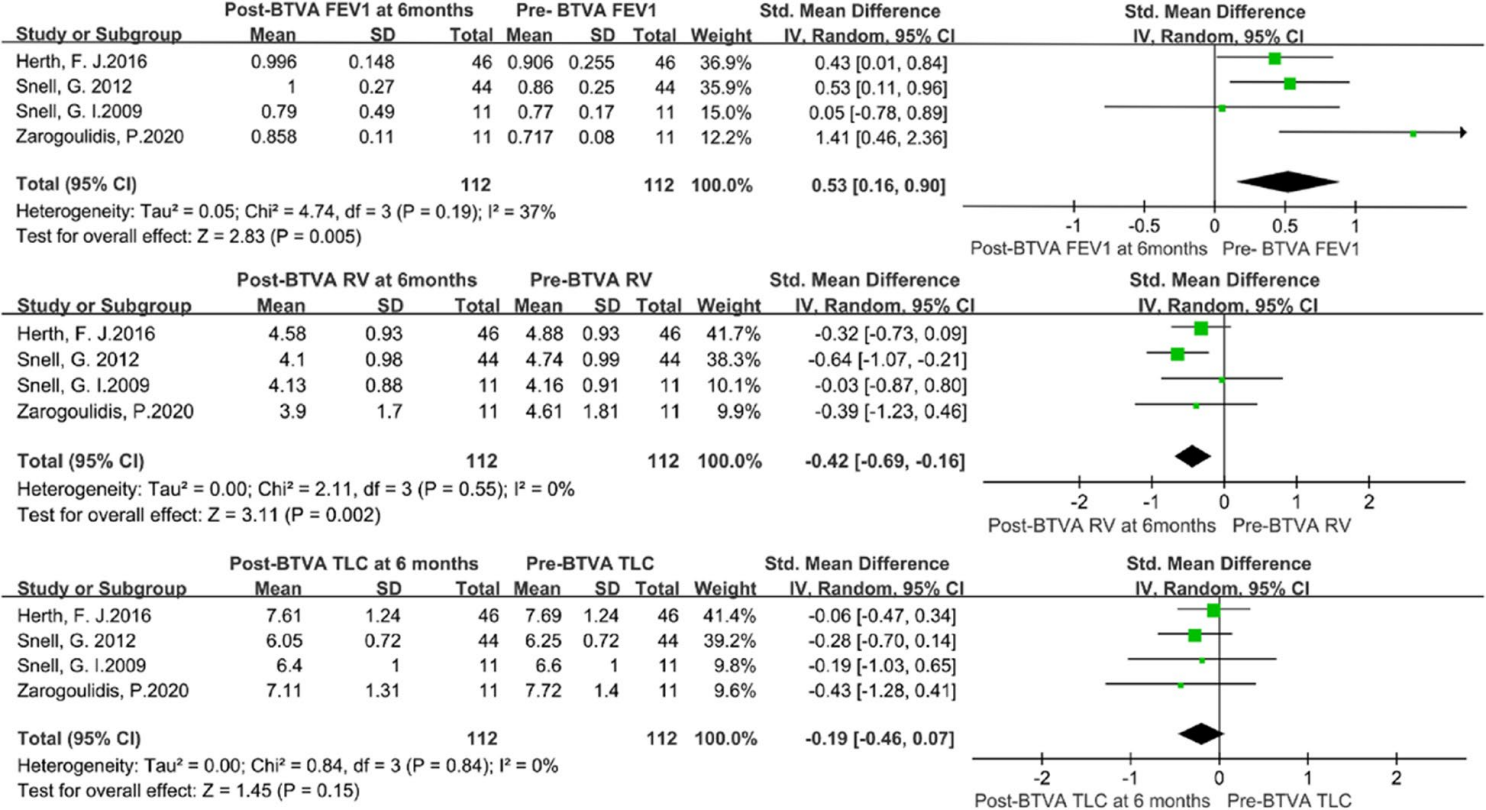
Meta-analysis and forest plot of all studies included about FEV1, RV and TLC between baseline and 6 months. Calculations based on a randomized-effects model. SMD, standardized mean difference; FEV1, forced expiratory volume in 1 s; RV, residual volume; TLC, total lung capacity

**Fig. 5:**
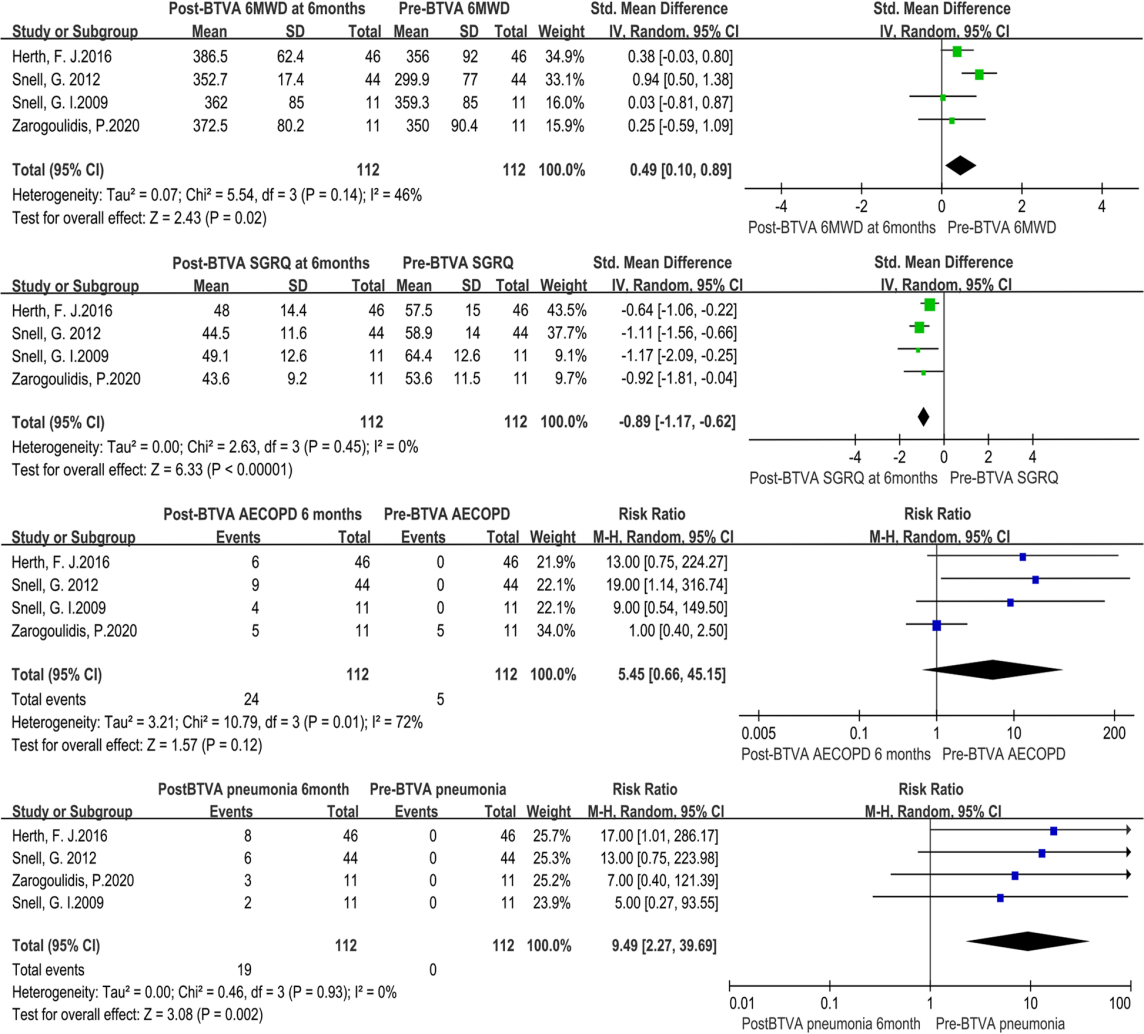
Meta-analysis and forest plot of all studies included about 6MWD, SGRQ, incidence of AECOPD and pneumonia between baseline and 6 months. Calculations based on a randomized-effects model. SMD, standardized mean difference; 6MWD, 6-min walk distance; SGRQ, St George’s Respiratory Questionnaire

The heterogeneity test revealed that there were no significant differences among individual studies (*P* > 0.05). And a randomized-effects model was used for the pooled analysis. Meta-analysis of the pooled effect showed that levels of FEV1 and SGRQ were significantly improved in patients with emphysema between 3 months vs. baseline (Figs. 2 and 3). Also, itshowed that levels of FEV1, RV, TLC, 6MWD and SGRQ were significantly improved in patients with emphysema between 6 months vs. baseline before BVTR being performed (Figs. 4 and 5).

Additionally, no significant FEV1, RV, TLC and SGRQ changes occurred from 3 to 6 months of follow-up except for 6MWD (Figs. S1 and S2). The magnitude of benefit was higher at 3 months compared to 6 months.

### Outcomes of complications

We pooled data related to complications in the included studies. The most common complications at 6 months were treatment-related COPD exacerbations (RR: 12.49; 95% CI: 3.06 to 50.99; *p* < 0.001) and pneumonia (RR: 9.49; 95% CI: 2.27 to 39.69; *p* < 0.001) (Fig. 5).

### Assessment of publication bias

**Fig. 6:**
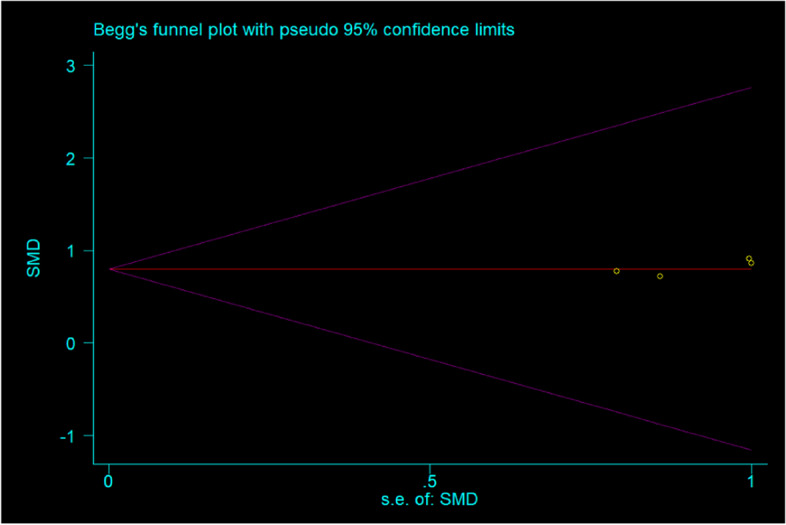
Funnel plots for assessing publication bias of studies included. SE, standard error; SMD, standardized mean Difference

**Fig. 7:**
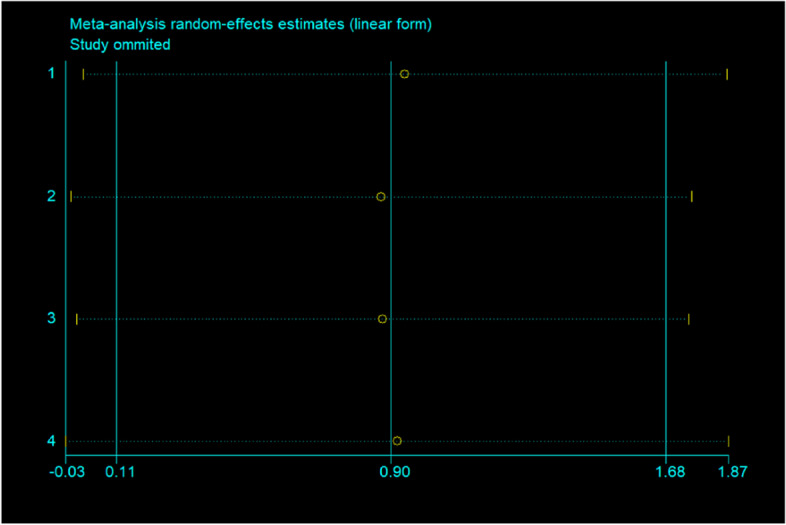
Sensitivity analysis of all studies included. Meta-analysis random-effects estimates (linear form)

The funnel plot (Fig. 6) suggested that small publication bias may exist. Additionally, Begg’s tests (*p* = 0.734) suggested no evidence to support publication bias in the meta-analysis. Furthermore, sensitivity analysis demonstrated that omitting any one of the studies at a time do not influence the overall result of the pooled analysis (Fig. 7).

## Discussion

To date, this is the first meta-analysis that systematically explored the impact of BTVA treatment on lung function and quality of life in patients with emphysema. Overall, compared with the parameters at baseline, there were dramatically apparent differences in FEV1, TLC, RV, 6MWD and SGRQ at 3 months. A remarkable finding from us was the short-term improvement of lung function and quality of life. And it remained to be determined about differences in BTVA-related improvements at long-term follow-up. And in line with previous data, the most common complications at 6 months wereCOPD exacerbations and pneumonia (Table 2).

**Table 2:** The summary of some studies in lung volume reduction

Study	Year	Sample size	Therapy duration	Treatment	Study design	Significant improvement indicators
Zhu, W [25]	2022	18	12 months	BTVA	non-RCT	FEV1 at 3 months, mMRC through the whole period
Zarogoulidis, P [17]	2020	11	9 months	BTVA	Observational	FEV1 at 6months, 6MWD and SGRQ at 6 and 9 months
Fiorelli, A [26]	2017	36	5 years	EBV	Observational	FEV1, FVC%, RV%, 6MWD and SGRQ at 12 months
Gompelmann, D [20]	2016	69	12 months	BTVA	RCT	FEV1 at 3 months, RV at 6 months
Herth, F. J [15]	2016	70	6 months	BTVA	RCT	FEV1, SGRQ at 6 months
Come, C. E [27]	2015	95	12 months	ELS	RCT	FEV1, mMRC, SGRQ at 3 months
Snell, G. I [14]	2012	44	6 months	BTVA	Open-label, single arm trial	FEV1 and RV at 6 months SGRQ, mMRC at 3 and 6 months
Shah, P. L [28]	2011	315	12 months	Airway bypass	RCT	FVC and mMRC at 6 months
Sciurba, F. C [29]	2010	321	12 months	EBV	RCT	FEV1 at 6 months
Snell, G. I [16]	2009	11	6 months	BTVA	Observational	No dramatic differences in FEV1, RV and 6MWD at 6 months

*EBV* Endobronchial valves, *ELS* emphysematous lung sealant, *6MWD* 6-min walk distance, *SGRQ* St George’s Respiratory Questionnaire, *mMRC* Medical Research Council Dyspnoea Score, *FEV1* forced expiratory volume in 1 s, *RCT* Randomized Controlled Trial, *BTVA* bronchial thermal vapor ablation

As the leading characteristic of COPD reflected by the abnormal and permanent enlargement of the airspaces distal to the terminal bronchioles, emphysema is associated with loss of elastic recoil and early airway closure during exhalation [30]. Attempts at LVR including LVRS and BLVR have been widely made, and there is real potential for them to achieve a satisfactory safety profile and efficacy on emphysema. LVRS could provide substantial long-term clinical benefit, unfortunately, it was not widely applied in patients with severe emphysema because of the high mortality and morbidity rates [6]. Indeed, the inter-lobar collateral ventilation may be responsible for the lower improvement relative to studies of LVRS [29]. Also, long-term results were illustrated after BLVR using endobronchial valves with maintained success for up to five years [26]. To the best of our knowledge, the endoscopic techniques used one-way valves, coils as well as thermal ablation, resulting in the collapse of overinflated lung segments and achieve benefits on emphysema similar to that of surgery [1]. A RCT of airway bypass in 315 patients with advanced emphysema illustrated an annual loss of FEV1 in the controls of approximately 6% decline [28]. An increase of FEV1 by 11.4% after 3 months and 18.9% after 6 months in the patients receiving an emphysematous lung sealants and significant improvements in 6MWD and SGRQ at 6 months were observed [27]. Notably, careful evaluation and selection of treatment strategy on the underlying patient with chronic obstructive disease was essential based on the advantage and shortcomings among all the approaches.

Different from other BLVR techniques,BTVA could increase elastic recoil by reducing the most compliant areas of lung, decompressing areas of healthy lung that allows for alveolar recruitment. Meanwhile, healthier segments should be preserved and allowed to expand after treatment to positively affect lung function, improve activity tolerance and preserve as much lung parenchyma as possible. Snell G et al. demonstrated that BTVA resulted in 48% lobar volume reduction at 6 months [14]. Additional data from a 12-month follow-up indicated that improvements relative to baseline continued to be observed, with the magnitude of benefit less than that documented at 6 months. However, HRCT lobar volume reduction was stable over 12 months [31]. Particularly, improvements from baseline to 6 months were similar for patients with GOLD stage III and IV disease, while improvements at 12 months were more robust in GOLD stage IV patients. The ceiling effect and differential contribution of small airways disease versus emphysema may be the explanation which lead to GOLD stage III patients having more regions of the lung that can have compensatory hyperinflation [31], which may be in agreement with our results. Despite of a numerical difference in the magnitude of effectiveness was observed from 3 to 12 months [14–17, 21, 25], BTVA was an available and well tolerated procedure for a great number of patients which are excluded from surgery or EBV for clinical issue.

Patient undergoing treatment of emphysematous segment within 12 weeks had an increase of 33% on FEV1. And significant differences in FEV1 and SGRQ at 6 and 12 months were also reported [16]. Also, STEP-UP study with BTVA targeting the more diseased segments of an upper lobe reported that around two-thirds of patients in the treatment group had a minimal clinically important difference in FEV1 or SGRQ at 3-month and 6-month follow-up visits [15], which was in line with our findings. Since emphysema of COPD is a progressive disease, BTVA provided a strategy of preserving lung tissue by allowing a personalized approach to the most diseased segments at the initial stage, resulting in the significantly clinical results at 6 months [32]. Additionally, Gompelmann et al. emphasized the selection criterion of patients undergoing BTVA that higher FEV1 and lower RV values would determine the therapeutic effect of the procedure [33]. Conversely, our meta-analysis of the pooled effect showed that only 6MWD was significantly improved between 3 and 6 months for BTVA except for the indicators of FEV1, RV, TLC, 6MWD and SGRQ. The magnitude of benefit was higher at 3 months compared to 6 months; We speculated that the initial effect for BTVA was significant related with the reduced hyperinflation and improvements in airflow, but there seems to be no obvious improvement at 6 months or longer-term follow-up. Possible explanations include compensatory hyperinflation of the contralateral lung, comorbidities and good adherence to prescribed respiratory medications.

Although TLC and RV were improved significantly in some studies as well as in our study [14, 15], no significant changes existed in CT-calculated TLC, the nontreated adjacent upper lobe remained of similar volume (2% increase) and that of lower lobe increased in volume by 24%. Narrowed or occluded segmental or subsegmental airways were described in all patients, with evident changes in most treated segmental airways [16]. Healthier segments should be preserved and allowed to expand after treatment to improve lung efficiency and preserve as much lung parenchyma as possible. Despite a reduction in the volume of the treated lung, the overall lack of change in CT lung volume is explained by the expansion of the adjacent nontreated lower lobes. Meanwhile, it should be noted that limited data on the performance of bronchoscopy in the airway for BTVA were observed.Part of treated segmental airways were noted to have localized pallor and a few had at least some visible tapering obstruction of a main segmental or subsegmental airway [16]. Therefore, an objective measure such as lobar volume reduction evaluated by CT and performance of bronchoscopy may be recommended in the estimation of improvements of BTVA.

Notably, the advantage of BTVA was to reduce emphysematous segments allowing for precise targeting of the more diseased segments [34]. Regarding serious adverse events, targeted therapy on the more destroyed areas may be superior to conventional treatment with a significant reduction on local inflammation. Although there was a significant increase of incidence in complications in our study, most data of the included studies only analyzed the complications at baseline and 6 months, rather than intermediate period, the conclusion needs caution. A short-term inflammatory response was led by the thermal energy, accomplished by the fibrosis along with atelectasis that occurs distally in the treated region and subsequent lobar volume reduction [14, 16]. Volume reduction following BTVA is a natural process that occurs gradually over a 4–6-week period. Actually, adverse events observed in the network meta-analysis had no significant difference among intrabronchial valve, endobronchial valve, lung volume reduction coils and BTVA [22]. BTVA seems to have a more favorable safety profile in comparison to lobar reduction. The explanation was speculative and it needs caution that the overall sample size is relatively small [14]. The localized inflammation appears to be responsible for acute exacerbation of COPD and pneumonia, which may be important concerns focused on by doctors, suggesting that some patients may be covered with spectrum antibiotics in order to reduce infection from our meta-analysis. Overall, adverse events leading to hospital admission in a 180-day follow-up period occurred in a minority of treated patients. The reaction seems to be aggravated within the first 2–4 weeks and gradually resolves within 8–12 weeks of BTVA. Radiographically, the targeted area will typically show infiltrates that could be indistinguishable from pneumonia [35]. Adverse events were associated with the volume of the treated lobe, so the dose chosen was in accordance with the balance between optimal benefit and acceptable risk [18]. A preclinical animal study applied higher doses than those in humans showing dose-dependent volume reduction [35]. Hence, segmental rather than lobar vapor treatment is recommended [33]. At present, patients with upper lobe predominant emphysema were studied to evaluate the overall benefit–risk in most researches except for one study enrolled a few patients in the lower lobes [17]. The effect of the target regions performed by BTVA in lower lobes needs explore.

Several limitations of our study should be admitted. Firstly, only patients with predominant upper lobe emphysema were enrolled. Whether the improvements could be extended to other segments by BTVA will require longer term follow-up to evaluate the overall benefit–risk [36]. Secondly, the follow-up period on benefits and safety observed following BTVA varied from 3 to 12 months. Indeed, the histopathologic response with inflammation followed by contraction fibrosis may be changed. Thirdly, the relatively small sample size has limited power to explore subgroups including targeted area of emphysema, severity of lung function and quality of life and adverse events at different stage. Heterogeneity maybe a phenotype that assists in assessment of patient suitable for BLVR [37]. Fourthly, only those studies published in English were pooled, and a few related studies published in other languages may be missed.

## Conclusions

Our meta-analysis provided clinically relevant information about the impact and safety of BTVA on predominantly upper lobe emphysema. Particularly, short-term significant improvement of lung function and quality of life occurred especially within the first 3 months. The dynamic differences in BTVA-related improvements and complications in long-term follow-up are needed to explore.

### Supplementary Information

**Additional file 1: Fig. S1.** Meta-analysis and forest plot of all studies included about FEV1, RV and TLC between 3 and 6 months. Calculations based on a randomized-effects model. SMD, standardized mean difference; FEV1,forced expiratory volume in 1 second; RV, residual volume; TLC, total lung capacity.

**Additional file 2: Fig. S2.** Meta-analysis and forest plot of all studies included about 6MWD and SGRQ between 3 and 6 months. Calculations based on a randomized-effects model. SMD, standardized mean difference; 6MWD, 6-minute walk distance; SGRQ, St George’s Respiratory Questionnaire.

## Data Availability

The datasets used and/or analyzed during the current study are available from the corresponding author on reasonable request.

## Abbreviations

FEV1: Forced expiratory volume in 1 s
RV: Residual volume
TLC: Total lung capacity
6MWD: 6-Minute walk distance
SGRQ: St George’s Respiratory Questionnaire
COPD: Chronic obstructive pulmonary disease
LVR: Lung volume reduction
BLVR: Bronchoscopic lung volume reduction
LVRS: Lung volume reduction surgery
RCTs: Randomized controlled trials
HRCT: High-resolution computed tomography
