# Supplementary material for: Impact of bronchoscopic thermal vapor ablation on lung volume reduction in patients with emphysema: a meta-analysis
Source: BMC Pulm Med. 2023 Oct 26;23:405. doi: 10.1186/s12890-023-02689-w (PMC10601098; doi:10.1186/s12890-023-02689-w)
Supplement: Supplementary file 2 — Additional file 2: Fig. S2. Meta-analysis and forest plot of all studies included about 6MWD and SGRQ between 3 and 6 months. Calculations based on a randomized-effects model. SMD, standardized mean difference; 6MWD, 6-minute walk distance; SGRQ, St George’s Respiratory Questionnaire. [file 12890_2023_2689_MOESM2_ESM.docx]

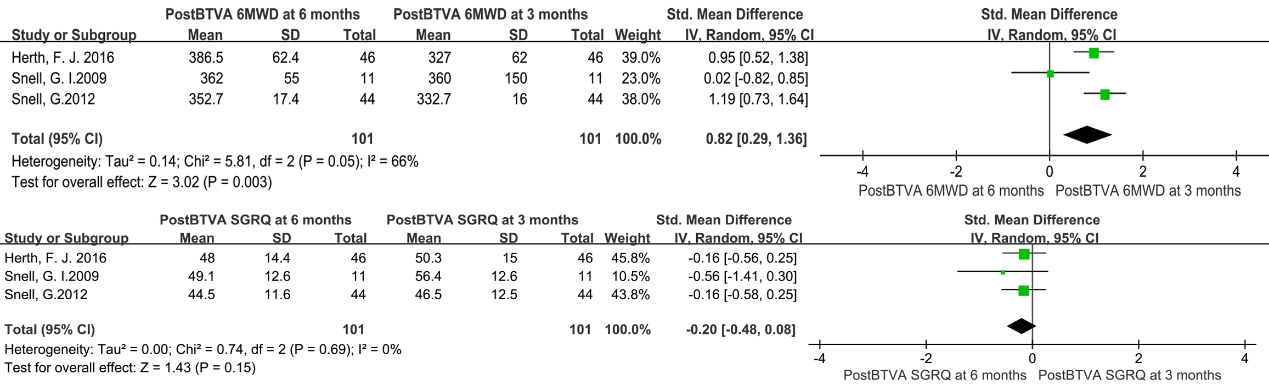


Fig. S2 Meta-analysis and forest plot of all studies included about 6MWD and SGRQ between 3 and 6 months. Calculations based on a randomized-effects model. SMD, standardized mean difference; 6MWD, 6-minute walk distance; SGRQ, St George’s Respiratory Questionnaire;
